# Partial prosthesis detachment early after open atrial transcatheter mitral valve replacement: could an artificial intelligence–based modified mitral valve model make the difference?

**DOI:** 10.1093/ehjimp/qyaf096

**Published:** 2025-07-30

**Authors:** Aureliano Ruggio, Gabriella Locorotondo, Andrea Campea, Riccardo Marano, Eleonora Moliterno, Francesca Graziani, Cristina Aurigemma, Faustino Pennestrì, Antonella Lombardo, Francesco Burzotta

**Affiliations:** Department of Cardiovascular Sciences, Fondazione Policlinico Universitario A. Gemelli IRCCS, Largo A. Gemelli 8, 00168 Rome, Italy; Department of Cardiovascular Sciences, Fondazione Policlinico Universitario A. Gemelli IRCCS, Largo A. Gemelli 8, 00168 Rome, Italy; Department of Cardiovascular Sciences, Fondazione Policlinico Universitario A. Gemelli IRCCS, Largo A. Gemelli 8, 00168 Rome, Italy; Department of Diagnostic Imaging, Oncological Radiotherapy and Hematology, Fondazione Policlinico Universitario Agostino Gemelli IRCCS, Rome, Italy; Department of Radiological and Haematological Sciences–Section of Radiology, Università Cattolica del Sacro Cuore, Rome, Italy; Department of Radiological and Haematological Sciences–Section of Radiology, Università Cattolica del Sacro Cuore, Rome, Italy; Department of Cardiovascular Sciences, Fondazione Policlinico Universitario A. Gemelli IRCCS, Largo A. Gemelli 8, 00168 Rome, Italy; Department of Cardiovascular Sciences, Fondazione Policlinico Universitario A. Gemelli IRCCS, Largo A. Gemelli 8, 00168 Rome, Italy; Department of Cardiovascular Sciences, Università Cattolica del Sacro Cuore, Rome, Italy; Department of Cardiovascular Sciences, Fondazione Policlinico Universitario A. Gemelli IRCCS, Largo A. Gemelli 8, 00168 Rome, Italy; Department of Cardiovascular Sciences, Università Cattolica del Sacro Cuore, Rome, Italy; Department of Cardiovascular Sciences, Fondazione Policlinico Universitario A. Gemelli IRCCS, Largo A. Gemelli 8, 00168 Rome, Italy; Department of Cardiovascular Sciences, Università Cattolica del Sacro Cuore, Rome, Italy

**Keywords:** mitral annular calcification, transcatheter mitral valve replacement, THV embolization, artificial intelligence, modified 3D mitral valve model

A 75-year-old woman with severe mitral annular calcification (MAC) and significant mitral regurgitation, considered unsuitable for either surgery or transcatheter mitral valve (MV) replacement (TMVR) (*Figure 1A–H*), underwent open-transatrial TMVR with a 29-mm Sapien-3 Ultra aortic transcatheter heart valve (THV) implantation in another institution. Five months later, partial prosthesis detachment at the lateral commissure and severe paravalvular leak (PVL) (*Figure 1I–L*; Supplementary data online, *Video S1*) resulted in cardiogenic shock.

**Figure 1:**
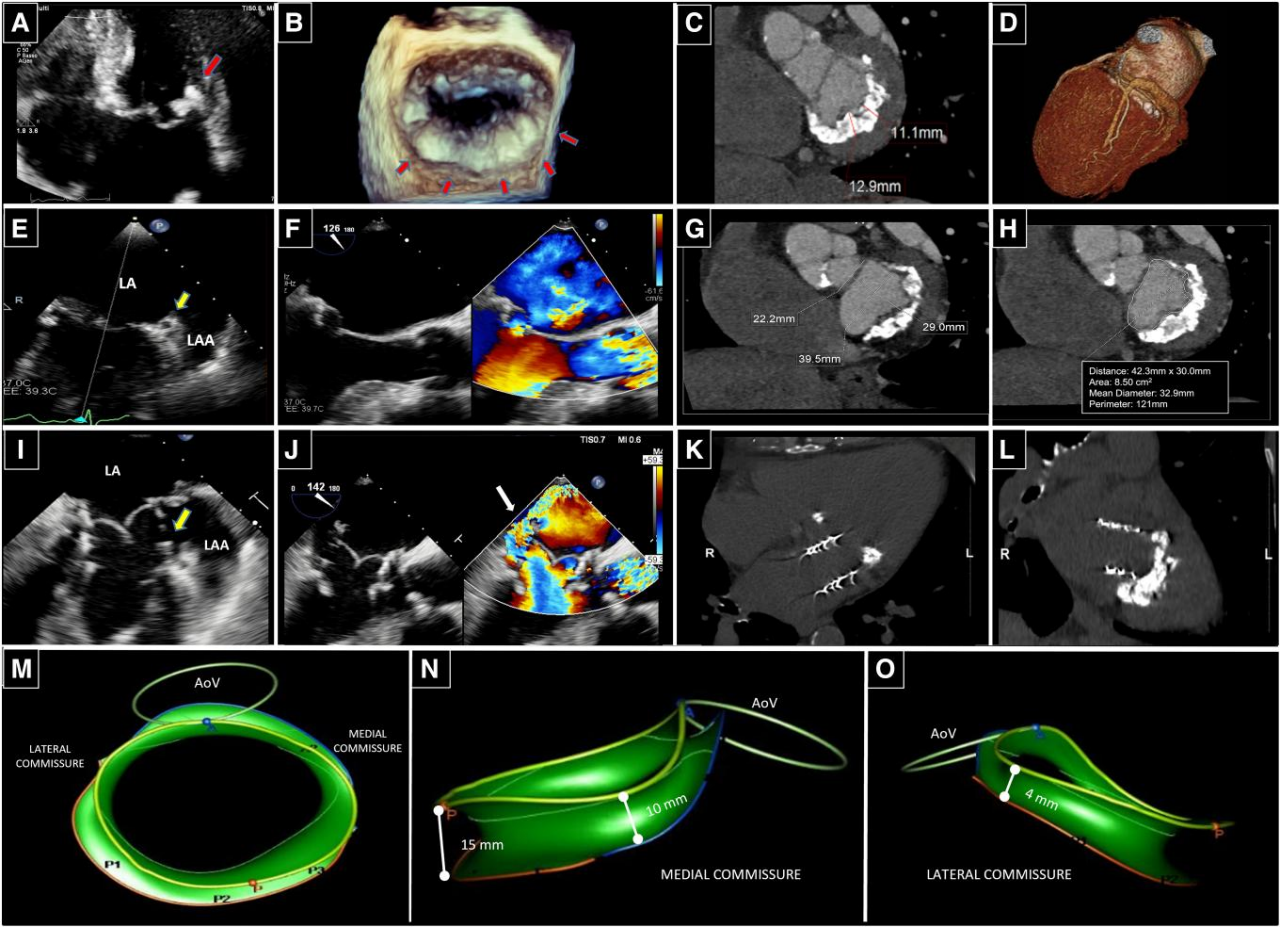
Multimodality imaging of MAC before (*A–H*) and after (*I–L*) surgery. Pre-operative transthoracic (*A*) and 3D transoesophageal echocardiography (TEE) *en face* (*B*) views showing an extensive MAC of the posterior mitral annulus (*arrows*). Pre-operative contrast-enhanced computed tomography (CT) short-axis (*C*, *G*, *H*) MPR images and volume rendering (*D*) showing *radial* calcium thickness > 10 mm, large mitral annulus diameters (29 mm anteroposterior, 39.5 mm bicommissural) and anatomical area (8.50 cm^2^), a nearly normal intertrigonal distance (22.2 mm), and calcium distribution > 270° with incomplete extension in the annular circumference (MAC score 8). TEE bicommissural (*E*, *I*) and long-axis (*F*, *J*) views showing pre-operative MAC (*E*, *F*) and post-operative severe PVL (*J*, *arrow*), extending from the posterior portion of the mitral annulus to the lateral commissure, near the circumflex artery (*I*, *arrow*). Long-axis (*K*, *L*) MPR images from the post-TMVR unenhanced CT showing device position, proximally to the MAC and protruding within the left atrium (LA). *3D-TEE-modified MV model* obtained by starting from the AI-based 3D MV annulus modelling application analysis, implemented into the TomTec software (TomTec-Arena, version TTA2.30.02, Philips, Italy; commercially available, standard across institutions) (*M–O*): by properly selecting planes and positioning reference points along the mitral annulus, at the MV leaflet coaptation, and at the aortic virtual basal ring, the AI-based software automatically provides a 3D MV reconstruction model which clearly shows planar or saddle-shape features, annular dimensions, ellipticity or sphericity, absolute displacement and displacement velocity, mitral-aortic angle, and leaflet extent. Once the automatic reconstruction is obtained, the model can be manually modified by moving the lines, which depict the leaflets, towards the ventricular side of the annular calcium, thus enveloping the entire calcium extent. In experienced hands, it takes about 30–60 min for the manual adjustments. Our model showed a nearly preserved saddle-shaped calcified mitral annulus along with an asymmetric *cranio-caudal* distribution of calcium thickness, greater at the posterior level (15 mm) and less represented at the lateral commissure (4 mm), where the PVL occurred. AoV, aortic valve; LA, left atrium; LAA, left atrial appendage; MPR, multi-planar reconstruction.

In patients with MAC and severe MV dysfunction, representing a high-risk subset strongly needing improvement in management, TMVR using balloon-expandable aortic THVs, through percutaneous or hybrid approaches, has emerged as a feasible option. Potential life-threatening complications, such as PVL and THV detachment, that occurred in our patient, can be predicted by a multi-slice computed tomography (MSCT)–based MAC score, which describes the circumferential and radial extent of MAC.

To assess anatomical contributors to failure, a retrospective analysis of our MAC case by an artificial intelligence (AI)–based *3D-modified MV model* (*Figure 1M–O*; Supplementary data online, *Video S2*) revealed anatomical details not evident on pre-operative MSCT scan: a nearly preserved saddle-shaped annulus (thus not ideally suited to a circular THV) and an asymmetric *cranio-caudal* calcium distribution along the mitral annular circumference (with a lower thickness at the lateral commissure, exactly where the PVL occurred). Both these 3D-derived parameters could represent potential imaging predictors of PVL and THV embolization.

Our case highlights a potential complementary role of an AI-enhanced 3D-modified MV model in the pre-operative screening of MAC patients by providing additional anatomical details as compared with CT-based MAC score alone, to personalize surgical planning.

Further studies are needed to confirm whether the integration of this model may improve patient selection, prediction of THV embolization risk, or even the futility of the procedure.

## Supplementary Material

qyaf096_Supplementary_Data

## Supplementary data

**Funding:** None declared.

**Data availability:** No new data were generated or analysed in support of this research.

## Lead author biography

Aureliano Ruggio, MD, is a cardiologist at the Department of Cardiovascular Sciences, IRCCS Foundation Agostino Gemelli Hospital, Rome, Italy, with expertise in cardiovascular imaging. His clinical focus is on 2D/3D transoesophageal echocardiography. He is fully certified in EACVI Adult Transthoracic and Transesophageal Echocardiography since October 2019. He is co-author of several publications on international peer-reviewed journals. He is a member of the Italian Society of Cardiology (SIC), Italian Society of Echocardiography and Cardiovascular Imaging (SIECVI) and EACVI Silver Member (ESC ID 869549).

